# Process Fingerprint in Micro-EDM Drilling

**DOI:** 10.3390/mi10040240

**Published:** 2019-04-11

**Authors:** Mattia Bellotti, Jun Qian, Dominiek Reynaerts

**Affiliations:** Department of Mechanical Engineering, KU Leuven, Member Flanders Make, 3001 Leuven, Belgium; mattia.bellotti@kuleuven.be (M.B.); jun.qian@kuleuven.be (J.Q.)

**Keywords:** electrical discharge machining, micro drilling, process monitoring, quality control

## Abstract

The micro electrical discharge machining (micro-EDM) process is extensively used in aerospace, automotive, and biomedical industries for drilling small holes in difficult-to-machine materials. However, due to the complexity of the electrical discharge phenomena, optimization of the processing parameters and quality control are time-consuming operations. In order to shorten these operations, this study investigates the applicability of a process fingerprint approach in micro-EDM drilling. This approach is based on the monitoring of a few selected physical quantities, which can be controlled in-line to maximize the drilling speed and meet the manufacturing tolerance. A Design of Experiments (DoE) is used to investigate the sensitivity of four selected physical quantities to variations in the processing parameters. Pearson’s correlation is used to evaluate the correlation of these quantities to some main performance and hole quality characteristics. Based on the experimental results, the potential of the process fingerprint approach in micro-EDM drilling is discussed. The results of this research provide a foundation for future in-line process optimization and quality control techniques based on machine learning.

## 1. Introduction

Micro electrical discharge machining (micro-EDM) drilling is a well-established non-contact thermal process for making small holes in electrically conductive materials, such as cooling holes in turbine blades and diesel injector nozzles [1,2]. In this process, the removal of material occurs through sequences of high-frequency electrical discharges within an electrically insulated gap between two electrodes. Deionized water or hydrocarbon oil are commonly applied as dielectric medium. Tubular tools of brass or copper material are used to increase the drilling speed [3,4].

Due to the complexity involved in the electrical discharging process, optimization of the processing parameters in micro-EDM drilling is an iterative and time-consuming process, which is often based on manual experimentation and user experience. Likewise, the complexity of the discharging phenomena does not facilitate the establishment of correlations between the applied processing parameters and the final hole quality. It follows that significant post-processing metrology efforts are often required for quality control and tolerance verification of micro-EDMed holes. Recent advancements in machine learning techniques offer new solutions for shortening and automating process optimization and allow for the creation of technology databases to reduce post-process metrology [5,6]. However, effective and reliable approaches for correlating the processing parameters and the relevant outputs in terms of efficiency of the drilling process and hole quality are needed for these purposes.

**Figure 1:**
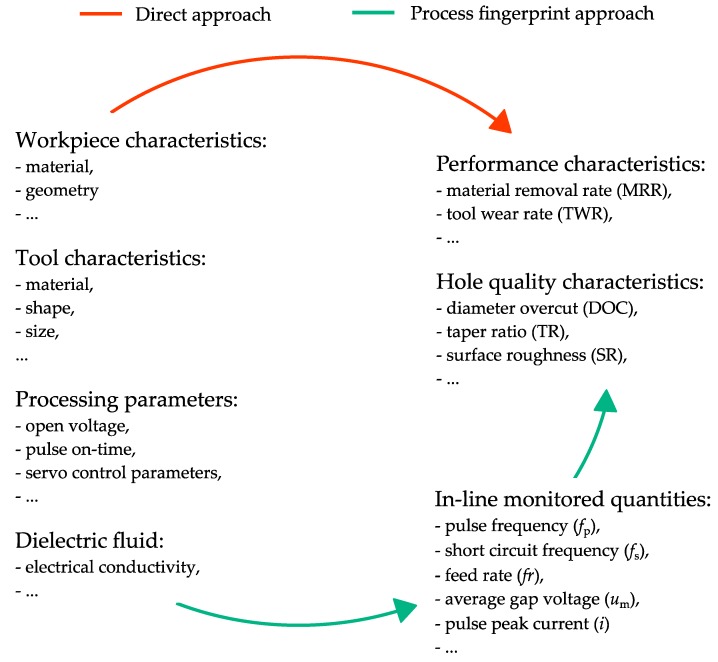
Following the process fingerprint approach, in-line monitored quantities can be used for optimizing the processing parameters and predicting the hole quality. This could reduce or even omit the post-processing metrology efforts required when following the direct approach.

Traditionally, a so-called ‘direct approach’ has been used for optimizing the processing parameters and predicting the hole quality in micro-EDM drilling. As shown in Figure 1, this approach focuses on establishing direct correlations between the processing parameters and the performance and quality characteristics. Extensive research has been conducted in recent years to establish such correlations. For instance, Lin et al. [7] used a response surface method to model the effects of different processing parameters on the material removal rate (MRR), tool wear rate (TWR), and diameter overcut (DOC) in micro-EDM of carbon tool steel. Jung et al. [8] and Ay et al. [9] used gray relational analysis for multiple performance optimization when processing stainless steel and Inconel 718 as a workpiece material. D’Urso et al. [10] defined two process windows representing the TWR and MRR as a function of the hole depth, considering various processing parameters and electrode materials. Jahan et al. [11] studied the influence of the process-energy parameters on the MRR and taper ratio (TR) in micro-EDM drilling of tungsten carbide. Suganthi et al. [12] used adaptive neuro-fuzzy inference system and artificial neural networks to predict the MRR, TWR, and surface roughness (SR) from the processing parameter settings. Although using direct correlations could be an effective solution for optimizing the processing parameters, no real-time feedback can be obtained during the drilling process to check whether any variation from the process conditions, under which the correlations were established, is occurring. Therefore, post-processing measurements are required to check the hole quality. Accurately performing these measurements could be tedious or, in some cases, not even possible. (e.g., small hole size or accessibility problems).

An alternative to the direct approach could be the process fingerprint approach, which attempts to find correlations between the performance and quality characteristics and measurable physical quantities that can be monitored and controlled in-line (Figure 1). In this context, the term ‘process fingerprint’ refers to what is left on the workpiece after the manufacturing process, considering the mechanism and dynamics of the material removal process. The physical quantities, which are strongly correlated to the process fingerprint and are in-line monitored and controlled, can be denoted as indicators for the process fingerprint. The process fingerprint has previously also been referred to as ‘process signature’ in the specific case of surface integrity or surface modification in manufacturing processes [13,14]. For example, Sealy et al. [15] identified energy process signatures for the surface integrity problem in hard milling. Klink [16] introduced the concept of process signatures from the point of view of part functionality and surface modification in electrochemical machining (ECM) and EDM.

Following the process fingerprint approach, real-time optimization algorithms and decision-making systems can be implemented to maximize the drilling rate and meet the manufacturing tolerance by keeping the in-line monitored quantities within a desired range. Hence, the time for reaching the optimal setting of processing parameters could be shortened and post-processing metrology efforts could be drastically reduced or even omitted. The applicability of the process fingerprint approach to some micro-manufacturing processes has been proved, such as for micro-milling [17] and micro-injection moulding [18]. However, despite its unique advantages, the approach has not currently been applied in micro-EDM drilling.

In order to shorten the process optimization time and reduce post-process metrology efforts in micro-EDM drilling, this paper explores the applicability of the process fingerprint approach by quantitatively studying the correlations between some selected physical quantities, and the main performance and hole quality characteristics. Four quantities that are conventionally used for in-line monitoring of the micro-EDM process are investigated as potential indicators of the process fingerprint. A Design of Experiments (DoE) is used as a screening methodology to analyze the sensibility of these quantities to variations in the processing parameters. A correlation study is performed to identify the quantities that are mostly correlated to the main outputs in terms of drilling efficiency (MRR and TWR) and hole quality (DOC and TR). The most suitable indicators to be considered for the process fingerprint are subsequently identified and discussed.

## 2. Materials and Methods 

### 2.1. Experimental Setup

**Figure 2:**
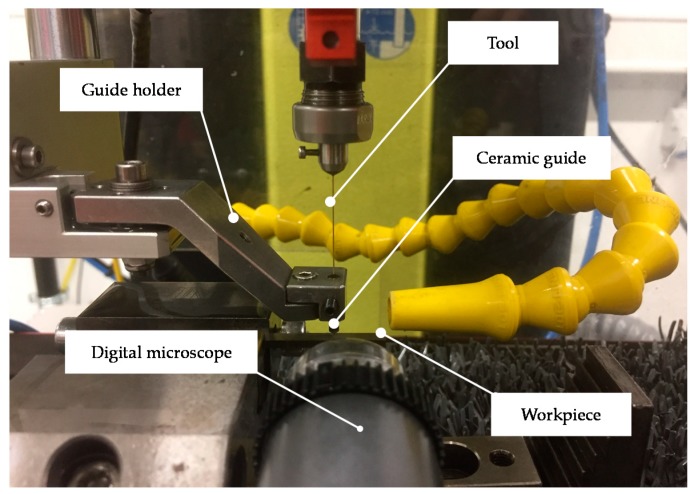
Experimental setup.

Micro-EDM drilling experiments were carried out on a SARIX^®^ SX-100-HPM (Sarix SA, Sant’Antonino, Switzerland) machine tool, which was equipped with the latest SARIX^®^ PULSAR pulse generator. A tool guiding system was used to reduce the run out of the tool when approaching the workpiece (Figure 2). This system consists of a ceramic tool guide and a guide holder. The tool guide was positioned at a distance of approximately 2 mm above the top surface of the workpiece.

Ti6Al4V was chosen as the workpiece material, since it is an alloy widely-used in biomedical and aerospace industries due to its excellent biocompatibility, high corrosion resistance, and good mechanical properties at high temperatures [19,20,21,22]. Commercial brass tubes provided by SARIX^®^ (outer diameter: 350 µm, inner diameter: 130 µm) were used as the tool electrode. Hydrocarbon oil (HEDMA^®^ 111) was applied as dielectric liquid. A combination of side and internal flushing was used. The flushing pressures were set to 0, 2, and 4 MPa respectively.

Series of through-holes were drilled into Ti6Al4V small plates of 2 mm thickness and 6 mm width. The plates were fixed as cantilevers into the workpiece holder to allow the tool to be fed through the hole outlet after the breakthrough. A digital microscope (Dino-Lite^®^ Edge AM4115ZT, AnMo Electronics Corporation, Hsinchu, Taiwan) was used to monitor the drilling process and interrupt the process as soon as a breakthrough was observed.

### 2.2. Process Monitoring

During the experiments, five variables were monitored in-line by the monitoring system embedded in the power generator of the SARIX^®^ machine. The main advantage of using this embedded system is that no external sensors or data processing systems are required. This considerably simplifies potential future implementations of the monitoring operations performed in this research into an industrial production environment.

The monitored variables were (i) *n*_p_: The number of normal discharge pulses, (ii) *n*_s_: The number of short circuits, (iii) *u*_m_: The average gap voltage, (iv) Δ*z*: The z-axis displacement of the ram of the machine tool, and (v) *t*: The drilling time. In particular, *n*_p_, *n*_s_, and *u*_m_ were recorded through the pulse generator system, Δ*z* was monitored by means of the z-axis encoder of the machine tool, and *t* was recorded using the clock of the computer of the control unit.

These five variables were synchronized and simultaneously updated at a rate of 100 Hz by the control unit of the machine, whereby a cumulative sum of each variable was stored. In order to monitor the variables at regular intervals, the drilling process was divided into steps of 50 µm in depth. This value refers to the nominal drilling depth as read by the z-axis encoder of the machine. Therefore, it does not correspond to the depth of the hole being drilled because of the longitudinal wear of the tool. At the end of each step, four quantities were computed in-line from the cumulative sums of *n*_p_, *n*_s_, *u*_m_, Δ*z* and *t*, which were subsequently zeroed. These quantities are:

- Pulse frequency (*f*_p_): The amount of normal discharge pulses per time unit,
- Short circuit frequency (*f*_s_): The amount of short circuits and arcs per time unit,
- Feed rate (*fr*): The speed at which the tool electrode is advanced into the workpiece, and
- Differential gap voltage (Δ*u*): The difference between the open voltage and average gap voltage.

**Figure 3:**
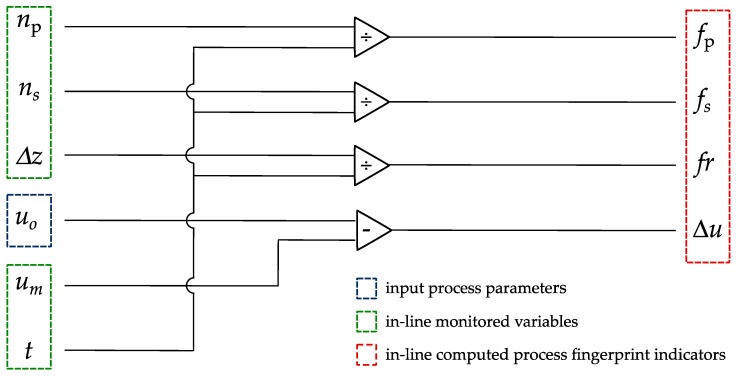
In-line calculation of the four quantities that are investigated as potential indicators for the process fingerprint of the micro electrical discharge machining (micro-EDM) drilling process (*n*_p_: Number of normal discharge pulses, n_s_: Number of short circuits and arcs, *u*_o_: Open voltage, *u*_m_: Average gap voltage, Δ*z*: Nominal drilling depth, *t*: Drilling time, *f*_p_: Frequency of normal discharge pulses, *f*_s_: Frequency of short circuits and arcs, *fr*: Feed rate, Δ*u*: Differential voltage).

Figure 3 provides a summary of the operations performed to compute these four quantities, which are considered as potential indicators for the process fingerprint in this research. While *f*_p_, *f*_s_, and *fr* are quantities that are conventionally monitored in micro-EDM operations for various purposes such as tool wear compensation [23,24] and breakthrough detection [25,26]. For the purposes of this study, the meaning of Δ*u* deserves a more detailed explanation. This Δ*u* quantity varies with the number of normal and abnormal discharge pulses, depending whether the abnormal discharges are short circuits or open circuits. In particular, Δ*u* is expected to approach zero when the discharging process is mostly in the state of open circuit, while it increases with the amount of normal discharge pulses or short circuits. Since short circuits and arcs are characterized by a lower discharge voltage than normal discharge pulses [27], the increase of Δ*u* is larger at the occurrence of short circuits and arcs rather than normal discharge pulses.

At the end of each drilling experiment, a comma-separated value (.csv) text file including the monitored values of *f*_p_, *f*_s_, *fr*, and Δ*u* at every 50 µm drilling step was automatically generated. The text files were later post-processed in order to identify the most suitable indicators for the process fingerprint.

### 2.3. Process Fingerprint Analysis

A post-process analysis was carried out to evaluate the correlation of the potential indicators for the process fingerprint to the performance and hole quality characteristics. A three-step analysis process was followed. First, the sensitivity of each indicator to changes of the processing parameters was investigated. Secondly, the correlation of each indicator to the MRR and TWR was evaluated. Thirdly, the correlations to the DOC and TR were computed. The Pearson’s correlation coefficient [28] was chosen to evaluate all correlations since it is a widely-used metric to evaluate the strength of the statistical relationship between two variables [29,30].

First, a DoE approach was adopted to investigate the sensitivity of the potential indicators for the process fingerprint to changes to the processing parameters. In particular, four processing parameters were varied in a two-level full factorial design. Four repetitions were carried out for each experiment, resulting in a total of 64 experimental runs. The selected processing parameters were the (A) pulse off-time, (B) open voltage, (C) servo adjustment factor, and (D) reference gap voltage. The servo adjustment factor is the proportional gain factor of the servo control loop. The processing parameters were chosen in order to include changes related either to the cycle time of the power supply system (A), to the energy input per discharge (B), or to the settings of the servo feed control system (C,D). The levels of the processing parameters were set so as to cover a wide process window. The selection was carried out according to the machine vendor’s recommendation and to previously reported experimental research involving a similar combination of tool and workpiece materials [4,22,25]. Table 1 is a summary of the experimental factors and their levels. 

The following processing parameters, not included in the DoE, were kept unchanged during the experiments: Pulse on-time – 4 µs, energy index – 301, current index – 70, tool rotation – 750 rpm, tool polarity – negative. According to the machine vendor, the energy index determines the energy and shape of the discharge pulses. The energy index 301 provides triangular pulses of medium-high energy content. When this energy index is selected, the current index can be used to regulate the pulse peak current.

Secondly, the correlation of each indicator to the MRR and TWR was analyzed in order to investigate the possibility of using the indicators of the process fingerprint for the purpose of optimizing the processing parameters. The MRR was calculated as the ratio between the volume of material removed from the workpiece (*V*_w_) and the total machining time (*T*), approximating the through-holes to a conical frustum:

(1) $$\mathrm{MRR}=\frac{V_{w}}{T}=\frac{\frac{\pi }{12}H(D_{in}^{2}+D_{out}^{2}+D_{in}\;D_{out})}{T}$$

where *H* is the thickness of the workpiece, and *D*_in_ and *D*_out_ are the inlet and outlet diameters of the holes, respectively, which were measured using a Werth VideoCheck HA coordinate measuring machine in optical mode. Similarly, the TWR was computed as the ratio between the volume of material removed from the tool (*V*_t_) and the total machining time (*T*). To compute such volume, the nominal inner (*d*_t_) and outer (*D*_t_) diameters of the tool were used, while the longitudinal wear was calculated by subtracting the workpiece thickness (*H*) to the monitored value of the drilling depth at the occurrence of breakthrough (*Z_b_*) as shown in Equation (2). 

(2) $$\mathrm{TWR}=\frac{V_{t}}{T}=\frac{\frac{\pi }{4}(D_{t}^{2}-d_{t}^{2})\;(Z_{b}-H)}{T}$$

Lastly, the DOC and TR were used to evaluate the quality of the drilled holes, since these are the two parameters that are mostly considered for assessing the geometrical characteristics of micro-EDMed holes [4,5,31,32,33,34]. The DOC was computed as:

(3) $$\mathrm{DOC}=D_{out}-D_{t}$$

while the TR was calculated as:

(4) $$\mathrm{TR}=\frac{D_{out}-D_{in}}{H}$$

The Pearson’s correlation coefficient (*r_p_*) was then calculated for all possible combinations between the indicators for the process fingerprint and the performance and hole quality characteristics. The *r*_p_ coefficient was computed as [28]:

(5) $$r_{p}=\frac{\sum_{i=1}^{64}(x_{i}-\bar{x})(y_{i}-\;\bar{y})}{\sqrt{\sum_{i=1}^{64}{\left(x_{i}-\bar{x}\right)}^{2}}\sqrt{\sum_{i=1}^{64}{\left(y_{i}-\bar{y}\right)}^{2}}}$$

where *x* and *y* are the vectors containing the datasets of the two quantities that are correlated, and $\bar{x}$ and $\bar{y}$ are their respective mean values. The average values of the indicators for the process fingerprint in each of the 64 experimental runs were considered. The value of *r*_p_ can vary between −1 and +1, which correspond to a perfect negative correlation and a perfect positive correlation, respectively. A value equal to 0 indicates the absence of a correlation between two considered data sets. Therefore, the closer the value of *r*_p_ to −1 or +1, the stronger the linear correlation between the two data sets.

## 3. Results and Discussion

### 3.1. Sensitivity to Changes to the Processing Parameters

**Figure 4:**
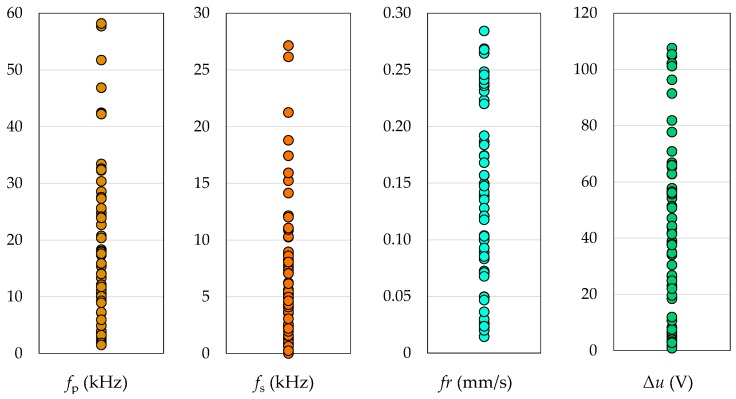
Plots of the data sets for the four potential indicators for the process fingerprint. Each data point corresponds to one of the 64 experimental runs.

**Figure 5:**
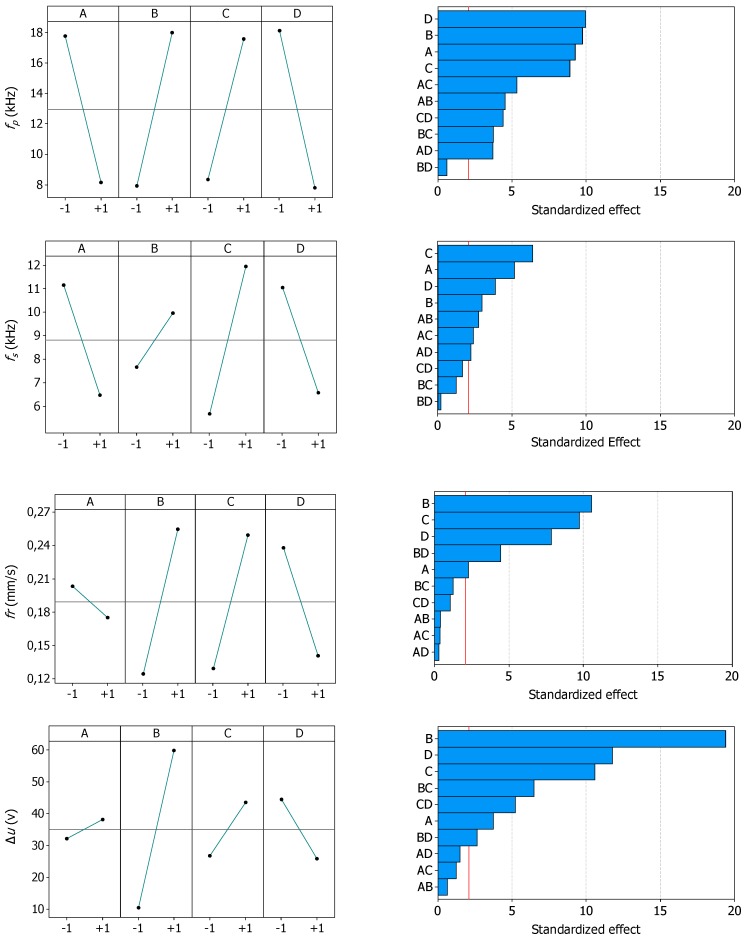
Influence of the four factors on the four potential indicators for the process fingerprint. The main effects plots (left column) and Pareto charts of the standardized effects (right column) are shown. The red vertical line in the Pareto charts corresponds to the significance level at 95% confidence level.

Figure 4 shows the data sets resulting from the 64 experimental runs. These data sets were used to analyze the sensitivity of the four potential indicators for the process fingerprint to changes to the processing parameters. For each indicator, the main effects plot and the Pareto chart of the effects are provided in Figure 5. The main effects plots can be used for analyzing the overall influence of each processing parameters on the indicators, while the Pareto charts show which effects are significant. This is of particular interest in this study since the indicators for the process fingerprint should be sensitive to variations to the process conditions, especially for the purpose of optimizing the processing parameters.

From the main effect plots it can be seen that *f*_p_ increased when a shorter pulse off-time, a higher energy amount per discharge pulse (i.e., higher open voltage), or more aggressive settings of the servo control system (i.e., higher servo adjustment factor or lower reference voltage) were applied. These effects are in line with previously reported results [24,31]. Therefore, they are not further discussed. Similar responses can be seen for *f*_s_ and *fr*, even though in both cases the effects of one factor (the open voltage for *f*_s_, and the pulse off-time for *fr*) were considerably less important than the ones of the other three factors. On the contrary, Δ*u* shows an increasing trend with the pulse off-time. This effect can be explained by the fact that a longer pulse off-time reduced the occurrence of both normal pulses and short circuits, thus increasing the amount of time spent in open-circuit state.

The Pareto charts reveal that all factors and second-order interactions, besides the one between the open voltage and reference voltage, had a significant influence on *f*_p_. This was deduced from the fact that the standardized effects were above the significance level. Moreover, *f*_p_ displayed similar standardized effects for the four factors. This means that *f*_p_ varied rather uniformly when changing different processing parameters. This is an appreciable characteristic for a process fingerprint indicator. Regarding the other three indicators, the Pareto charts show that *f*_s_, *fr*,,and Δ*u* were all sensible to changes to the single processing parameters, but not to most of the second-order interactions. In particular, *fr* was sensible to only one second-order interaction, i.e., the interaction between the open voltage and reference gap voltage. Furthermore, unlike *f*_p_, it can be noticed that *f*_s_, *fr*, and Δ*u* did not display a limited variability of the standardized effects. For instance, Δ*u* was highly sensible to changes to the open voltage, and *fr* to changes to the servo control parameters.

Based on the Pareto charts, it can be concluded that *f*_p_, *f*_s_, *fr*, and Δ*u* could be suitable quantities to be considered as indicators for the process fingerprint of the micro-EDM drilling process. This conclusion is drawn since the four indicators were at least sensible to changes to the single processing parameters. Nevertheless, thanks to more uniform sensitivity properties to changes to the processing parameters, *f*_p_ was the quantity that shows the highest potential.

### 3.2. Correlation with Performance Characteristics

**Figure 6:**
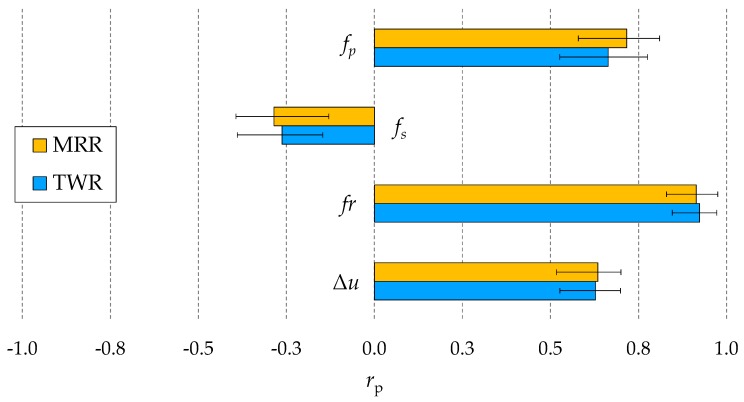
Pearson’s correlation coefficients (*r*_p_) of the four potential indicators for the process fingerprint with respect to the material removal rate (MRR) and tool wear rate (TWR). Mean values and ranges of the four experimental repetitions are shown.

The correlation coefficients of the potential indicators for the process fingerprint to the MRR and TWR are shown in Figure 6. It is evident that the correlation coefficient of *f*_s_ was significantly lower than the others. The reason for this can be found in the fact short circuits do not contribute to material removal in a predictable manner, as do normal discharge pulses. Short circuits are normally considered to be harmful to the drilling speed [35]. This explains why the correlation coefficients of *f*_s_ were negative, while the correlations coefficients of *f*_p_ were positive. Besides *f*_s_, the other three indicators displayed positive and relatively good correlations with the MRR and TWR. This indicates that maximization of *f*_p_, *fr*, or Δ*u* could be a viable way to increase the MRR, while minimization of one of these quantities could be pursued to reduce the TWR. The fact that the values of the *r*_p_ coefficients of *fr* were higher than the ones of *f*_p_ and Δ*u* suggests that *fr* could be the most suitable quantity to be monitored in-line for optimizing the processing parameters with respect to performance characteristics. Therefore, a linear regression analysis was carried out to further investigate the correlation of *fr* with the MRR and TWR. 

**Figure 7:**
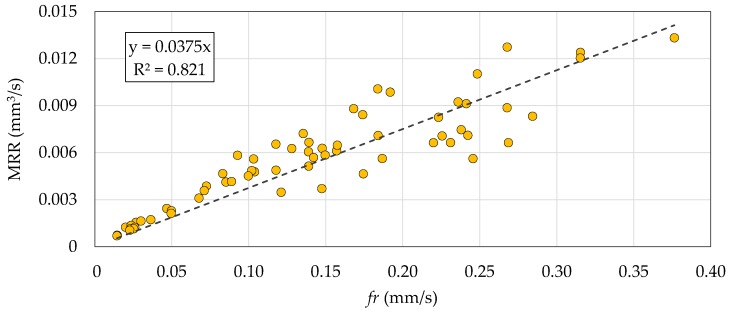
Correlation plot of the feed rate (*fr*) against the MRR. Each data point corresponds to one of the 64 experimental runs. The dashed line is the linear regression line.

**Figure 8:**
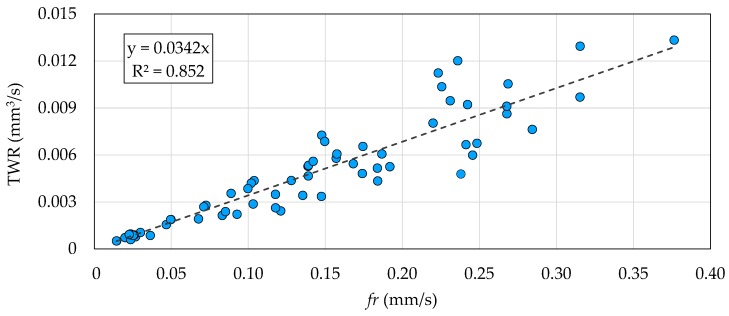
Correlation plot of *fr* against the TWR. Each data point corresponds to one of the 64 experimental runs. The dashed line is the linear regression line.

Figure 7 and Figure 8 show the correlation plots of *fr* against the MRR and TWR. A limited dispersion of the data points around the linear regression lines can be observed in both cases. This means strong positive linear correlations existed between *fr* and the MRR and TWR. The strength of the correlations is highlighted by the R^2^ coefficients above 0.8. Despite the better sensitivity property of *f*_p_, the result of this correlation analysis can be used to conclude that *fr* is the best quantity to be considered as process fingerprint for the purpose of optimizing the processing parameters.

**Figure 9:**
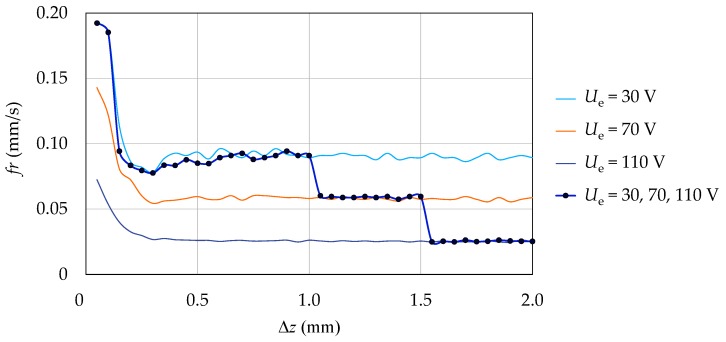
A comparison of the evolution of *fr* during four different drilling experiments. In three experiments the reference voltage (*U*_e_) is unvaried, while in the other experiment *U*_e_ is varied at steps of 0.5 mm in depth after touch-in (first 0.5 mm in depth). The data points correspond to the in-line monitored values of *fr* during the latter experiment. The other parameters are unvaried during the four experiments. In particular, the levels of the factors are: A = +1, B = −1, C = −1.

A possible algorithm to optimize the processing parameters could be based on a stochastic optimization technique [36]. However, in comparison with a previous application of this technique [6], the processing parameters could be varied during the drilling process of a single hole when following an approach based on in-line monitoring of *fr*. This would reduce the amount of time spent in each optimization iteration. As shown in Figure 9, after the touch-in stage, *fr* reached a stable value rather quickly when varying the processing parameters during the drilling process. This suggests that different settings of the processing parameters could be tried at regular steps during the drilling process (e.g., steps of 0.5 mm in depth). The measurement cycles to estimate the tool wear at the end of each drilling process could also be avoided. In this way, the time to reach the optimal setting of the processing parameters would be significantly reduced.

### 3.3. Correlation with the Hole Quality

**Figure 10:**
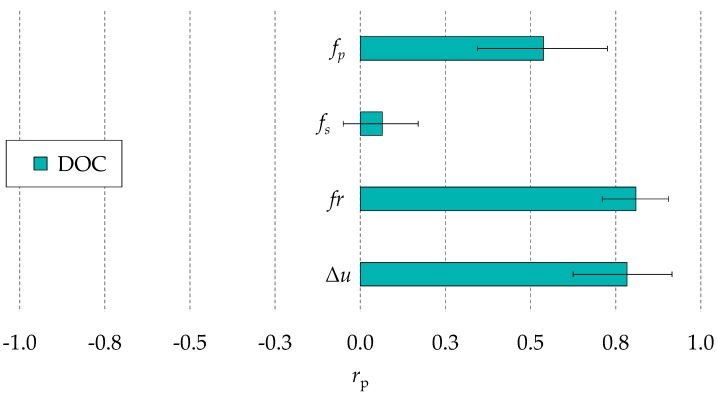
Pearson’s correlation coefficients (*r*_p_) of the potential indicators for the process fingerprint with respect to the diameter overcut (DOC). Mean values and ranges of the four experimental repetitions are shown.

Figure 10 shows the values of the correlation coefficients of *f*_p_, *f*_s_, *fr*, and Δ*u* to the DOC. Since short circuits and arcs do not contribute to material removal in a predictable manner, no correlation exists between *f*_s_ and the diameter of the hole inlet. On the contrary, the other three process fingerprint candidates showed a relatively good correlation with DOC. A possible reason can be that the DOC depends on the amount of discharge pulses occurring on the side of the tool and on the size of the discharge gap, which is dependent on the discharge energy. The main effect plots in Figure 5 confirm that *f*_p_*, fr* and Δ*u* tended to increase when the discharge energy increased or when the settings of the servo control system were more aggressive, a condition which might favour the ignition of discharges on the tool side. The fact that the *r*_p_ coefficient of *f*_p_ was lower than the ones of *fr* and Δ*u* could imply that a higher frequency of discharges does not correspond to a higher probability of discharges on the side of the tool. Both *fr* and Δ*u* could be used as indicators for the process fingerprint for in-line control of the DOC, since the mean values of *r*_p_ are above 0.8 for both quantities. Nevertheless, *fr* can be considered most suitable for controlling the DOC, considering that it displays a more limited variation of the *r*_p_ coefficient among the four experimental runs.

**Figure 11:**
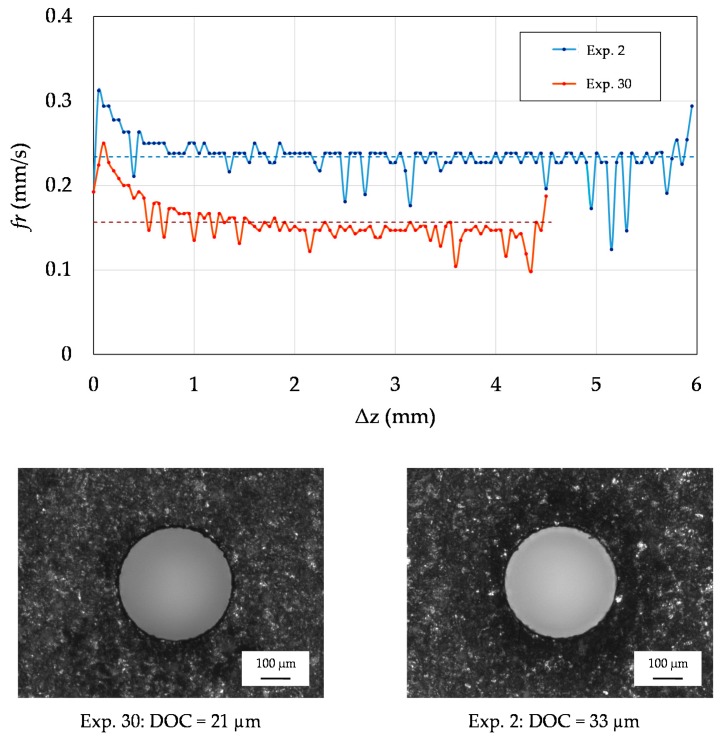
Evolution of *fr* during drilling. The data points represent the in-line monitored values of *fr*, while the dashed lines correspond to the average values. The evolution of *fr* and the hole inlets relative to two experimental runs are shown. Levels of the factors in Experiment 2: A = −1, B = +1, C = −1, D = +1. Levels of the factors in Experiment 30: A = −1, B = −1, C = +1, D = −1.

Figure 11 depicts the evolution of *fr* during the two drilling experiments resulting in the minimum and maximum DOC. The trends of the two sets of in-line monitored data points are not overlapping each other, and a relevant difference in the average values of *fr* can be observed. This confirms the suitability of using the average value of *fr* as the output of a real-time algorithm for controlling the DOC during the drilling process.

**Figure 12:**
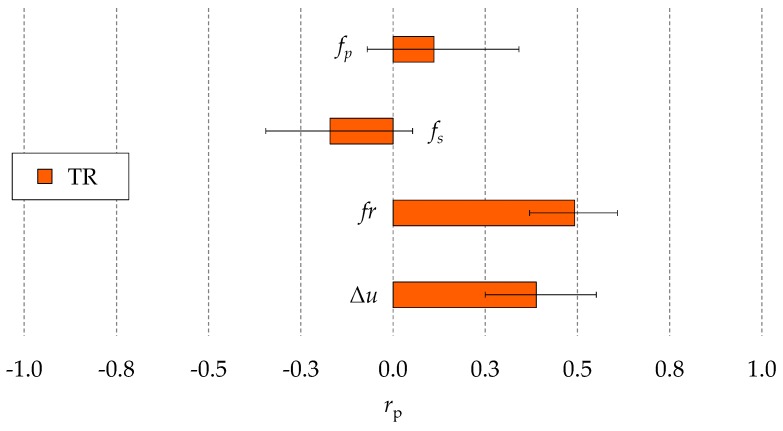
Pearson’s correlation coefficients (*r*_p_) of the potential indicators for the process fingerprint to the taper ratio (TR). Mean values and ranges of the four experimental repetitions are shown.

Figure 12 shows the correlation coefficients of the potential indicators for the process fingerprint to the TR. It can be clearly noticed that the mean values or the *r*_p_ coefficients of *f*_p_ and *f*_s_ were extremely low (less than 0.25). Moreover, the ranges of these coefficients considering the four experimental runs were relatively wide and around the zero point. Hence the correlations of *f*_p_ and *f*_s_ to the TR were weak. The *r*_p_ coefficient of Δ*u* was also low, while *fr* displayed a relatively good correlation to the TR. This means that *fr* is the only quantity that can be monitored in-line for controlling the taper of the micro holes among the four quantities considered in this research. The hole taper was mainly determined by the shape modifications of the tool tip due to the occurrence of discharge pulses on the side of the tool electrode. Therefore, the value of the correlation coefficients analysis suggest that the discharge pulses were more likely to occur on the side of the tool when the average feed rate of the tool electrode increased rather than when the total number of discharges was higher. This also explains why the correlation of *fr* and Δ*u* to DOC were higher than the one of *f*_p_.

It should be highlighted that a constant hole aspect ratio was considered in this research. However, the experimental results of Ali et al. [33] showed that the DOC and TR of micro-EDMed holes increase with the increase of the hole aspect ratio at almost the same rate. Therefore, similar correlation trends as the ones identified here can be expected when drilling micro holes of different aspect ratios. Overall, the process fingerprint approach is universal. Although this research has focused on a specific combination of tool and workpiece material, the approach can be applied in different process conditions.

## 4. Conclusions

This research evaluated the applicability of the process fingerprint approach for reducing post-process metrology and shortening process optimization in micro-EDM drilling. In contrast with traditional approaches that focuses on establishing direct correlations between the processing parameters and the hole quality characteristics, this approach is based on in-line monitoring of a few selected physical quantities. The correlation of four different physical quantities to some of the main performance and hole quality characteristics (i.e., the material removal rate, tool wear rate, diameter overcut, and taper ratio) has been investigated within a wide process window. 

The experimental results have shown that the average feed rate of the tool electrode displays a good correlation to the considered characteristics. Thus, it can be used as an indicator of the process fingerprint of micro-EDM drilling. This means that a real-time decision-making system could be implemented for optimizing the processing parameters or meeting a geometrical tolerance by keeping the average feed rate within a desired range during the drilling process. 

The results of this research enable new solutions for automatic optimization of the processing parameters and in-line quality control using machine learning. Future work should focus on determining suitable control algorithms for these purposes and extending the approach to other relevant hole quality characteristics, such as the surface roughness and recast layer thickness.

## Figures and Tables

**Table 1:** Design of Experiments (DoE): factors and levels.

Factor	Process Parameter	Unit	Level −1	Level +1
A	Pulse off-time	µs	5	20
B	Open voltage	V	80	160
C	Servo adjustment factor	–	20	80
D	Reference voltage	V	30	70
